# Medication Adherence of Vietnamese Outpatients with Chronic Diseases during the COVID-19 Pandemic

**DOI:** 10.3390/tropicalmed7060101

**Published:** 2022-06-13

**Authors:** Suol Thanh Pham, Cuong Van Dam, Chu Xuan Duong, Nghi Huynh Phuong Duong, Xuyen Thi Kim Nguyen, Han Gia Diep, Nguyet Kim Nguyen, Duyen Thi Nhan Le, Trang Thi Nhu Nguyen, Tu Thi Cam Le, Thao Thi Thanh Nguyen, Henri van Asten, Thang Nguyen

**Affiliations:** 1 Department of Pharmacology and Clinical Pharmacy, Can Tho University of Medicine and Pharmacy, Can Tho City 900000, Vietnam; ptsuol@ctump.edu.vn (S.T.P.); dxchu@ctump.edu.vn (C.X.D.); dgiahan6002@gmail.com (H.G.D.); kimnguyetnguyen@gmail.com (N.K.N.); ltctu@ctump.edu.vn (T.T.C.L.); 2 Faculty of Medicine, Nam Can Tho University, Can Tho City 900000, Vietnam; dvcuong@nctu.edu.vn (C.V.D.); nguyenxuyen2697@gmail.com (X.T.K.N.); 3 Department of Organization and Administration, Ninh Kieu District Medical Center, Can Tho City 900000, Vietnam; dgnghi1610@gmail.com; 4 Office of Science and Technology—External Relations, Can Tho University of Medicine and Pharmacy, Can Tho City 900000, Vietnam; ntntrang@ctump.edu.vn; 5 Faculty of Public Health, Can Tho University of Medicine and Pharmacy, Can Tho City 900000, Vietnam; nttthao@ctump.edu.vn; 6 Nijmegen Institute for International Health (NIIH), Radboud University Nijmegen Medical Centre, 6525 XZ Nijmegen, The Netherlands; h.vanasten@radboudumc.nl

**Keywords:** medication adherence, COVID-19, GMAS, 5K message, Vietnam

## Abstract

The purpose of this study was to determine the medication adherence of outpatients with chronic diseases and the association between both patient attitudes and preventive practices regarding COVID-19 and their medication adherence. We performed a cross-sectional study in Vietnam. Medication adherence was determined using the translated and validated Vietnamese version of the General Medication Adherence Scale (GMAS). Patient attitudes and preventive practices regarding COVID-19 were measured using the 5K message of the Vietnam Ministry of Health (facemasks, disinfection, distance, no gatherings, health declarations). The associations between patient characteristics and medication adherence were determined by multivariable regression. The study included 1852 outpatients, and 57.6% of the patients adhered to their medications. Patients who recognized the pandemic’s obstruction of medical follow-ups (OR = 1.771; 95%CI = 1.461–2.147; *p* < 0.001), who applied ≥2 preventive methods (OR = 1.422; 95%CI = 1.173–1.725; *p* = 0.001), who were employed (OR = 1.677; 95%CI = 1.251–2.248; *p* = 0.001), who were living in urban areas (OR = 1.336; 95%CI = 1.090–1.637; *p* = 0.005,) who possessed higher education levels (OR = 1.313; 95%CI = 1.059–1.629; *p* = 0.013), or who had ≤2 comorbidities (OR = 1.293; 95%CI = 1.044–1.600; *p* = 0.019) were more likely to adhere to their medications. The adherence percentage for outpatients with chronic diseases was quite low during the pandemic. Patients who did not recognize the COVID-19 pandemic’s obstruction of medical follow-ups or who had poor preventive practices were less likely to adhere to medications. Healthcare providers should pay more attention to these groups to achieve desired treatment outcomes.

## 1. Introduction

At the end of 2019, the International Committee on Taxonomy of Viruses (ICTV) named an unknown etiology of pneumonia that originated in Wuhan (China) severe acute respiratory syndrome coronavirus 2 (SARS-COV-2) [1]. Since COVID-19 first appeared, nearly 210 million people have been infected, and more than 4 million deaths have been reported worldwide. In South East Asia alone, more than 40 million confirmed cases have been recorded [2]. In Vietnam, the new community outbreaks at the end of January continued to evolve fast. A total of 4034 locally acquired COVID-19 cases and 12 deaths were reported as of 30 May 2021. Significantly, the period from March to May recorded more than 3000 locally transmitted cases of COVID-19 [3,4]. In response to the pandemic’s complexity, the governments of Vietnam and other countries have instigated strict directives to prevent the spread of COVID-19. Those directives include limiting movement, keeping a distance when interacting with others, and maneuvering medical staff to the front lines of the COVID-19 outbreak. These directives are necessary to alleviate the pandemic outbreak but possibly affect the treatment of patients, especially those with chronic diseases who require the use of medication and regular medical follow-ups. The study of Ethiopian public health facilities by Shimels et al. (2020) showed that 72% of patients with diabetes and hypertension were not compliant with medication. Moreover, before the COVID-19 pandemic, the adherence percentage for antihypertensive medication was 50% [5,6], and the figures for diabetes medication ranged from 51.3% to 76% [7,8].

Medication non-adherence is a challenging issue that worsens clinical outcomes and increases healthcare costs, especially those related to chronic diseases [9]. Various factors associated with medication non-adherence have been reported in previous studies, including living in rural areas, having multiple comorbidities, history of metabolic disorders, low family income, side effects of medications, etc. [10,11,12]. However, the factors listed above might vary among countries depending on the characteristics of each country or region. In addition, the anxiety of patients during the pandemic [13] was reported to be a reason for the failure to attend medical follow-ups and to adhere to medication according to treatment guidelines. The spreading of the COVID-19 pandemic might affect the attending of follow-ups and the receiving of drugs for patients with chronic diseases, and this could raise the risk of infection in outpatient clinics. Understanding the impacts of the pandemic on the medication adherence of patients with chronic health conditions helps the government and healthcare providers to implement appropriate plans to support patients for continuous monitoring and treatment, which, in the context of the pandemic, is extremely important for the long-lasting achievement of the desired treatment outcomes and increasing patient quality of life for those with chronic diseases. 

This study aimed to determine the prevalence of medication adherence, the perceived impacts of the pandemic, and the underlying social factors of patients with chronic diseases who were treated in outpatient clinics during the COVID-19 pandemic.

## 2. Materials and Methods

### 2.1. Study Designs and Sampling Methods

We conducted a cross-sectional study on outpatients having chronic diseases in some provinces of Southern Vietnam, including Can Tho, Ca Mau, Tay Ninh, Kontum, and Kien Giang, from March to May 2021. The selection criteria were: patients with chronic diseases who had been treated for at least 6 months and had medical follow-up visits during office hours at outpatient clinics. The sample size was calculated using the single proportion formula with the estimated medication adherence rate of 0.655, type I error probability of 5%, and a confidence level of 95%; the design effect was 5 [14]. An additional 5% were selected to allow for the loss of some participants due to medical follow-up; thus, there were 1852 patients in this study.

### 2.2. Data Collection and Analysis

Participants were selected through a convenience sampling method. The data were collected at the current workplaces of students studying at Can Tho University of Medicine and Pharmacy. The patients who came to the outpatient clinics at the time of the research were invited to a face-to-face interview through the prepared questionnaire. General information (name, age, job, etc.) and the General Medication Adherence Scale (GMAS) were included in the questionnaire. The collectors were previously trained to collect the data.

Medication adherence was measured and assessed through the GMAS version adjusted and validated in Vietnam [14]. The GMAS scale contains 11 questions, scored from 0 to 3 points for each one, and surveys 3 aspects of drug adherence: (1) patient behavior, (2) additional disease and pill burdens, (3) financial constraints. Aspects of medication adherence aspects were categorized into 5 levels ranging from high to poor. The scores for the aspects of drug adherence were classified as below:


**High**

**Good**

**Partial**

**Low**

**Poor**
Patient behavior11–15 points11–12 points8–10 points5–7 points0–4 pointsAdditional disease and pill burdens11–12 points9–10 points6–8 points4–5 points0–3 pointsFinancial constraints6 points5 points3–4 points2 points0–1 points

The total score of the scale was classified into two groups: non-adherence (0–26 points) and adherence (27–33 points) [14,15]. The proportion of medication adherence was determined by the number of compliant patients divided by the total number of patients at the time of the study.

The impact of the COVID-19 pandemic on medication adherence was assessed through self-reported attitudes and practices. Patient perspectives included their opinions on the pandemic’s impacts which drive them to continue their treatments and follow-up care. During medical follow-up visits, patient practices were surveyed on their adherence to the 5K message (wearing a facemask, disinfecting, keeping distance, not gathering, and carrying a health declaration). The clarification for the 5K message is shown in Appendix A. Therefore, we also investigated the combination of measures (0–1 measure versus ≥ 2 measures).

### 2.3. Statistical Analysis

Descriptive statistics summarize categorical (proportions and frequencies) and continuous variables (means, standards deviations). The chi-square test, with odds ratios (OR) and 95% confidence intervals (CIs), was applied to assess the relationships between medication adherence and population characteristics and the impacts of the COVID-19 pandemic. The variables having univariable results with *p*-values ≤ 0.1 were analyzed by multivariable logistic regression with the backward Wald method, which aimed to evaluate the impact of the variables on medication adherence. The Hosmer–Lemeshow test assessed the goodness of fit for logistic regression models. Data were entered using Microsoft Excel and were processed using SPSS version 22.0 (IBM Corp., New York, NY, USA). The outcomes were considered statistically significant if the *p*-values were < 0.05.

### 2.4. Ethics Approval

Ethical approval for this study was obtained from the Medical Ethics Councils of Can Tho University of Medicine and Pharmacy and the provincial hospital (1541/QD-DHYDCT). Participants were informed of the purpose and procedures of the study and voluntarily signed an informed consent form.

## 3. Results

### 3.1. Study Population Characteristics

A total of 1852 patients were selected (mean age = 60.12 ± 12.91 years, female sex = 1011, 54.6%). Most patients were not employed (84.0%) and had high school or higher education levels (65.4%). Cardiovascular and endocrine disorders were the most common diseases (43.5% and 32.8%, respectively). The mean duration of disease was 7.43 ± 6.06 years. The mean duration of treatment was 7.21 ± 6.01 years. The majority (72.8%) of patients had 0–1 comorbidity (Table 1).

54.6% of patients admitted that the COVID-19 pandemic affected their treatment follow-up visits. Additionally, 71.7% of patients felt worried during their visits to outpatient clinics. Regarding the 5K message, wearing facemasks (95.3%) and disinfecting (46.4%) were the most applied measures by outpatients during medical follow-up visits. The proportion of patients using at least 2 measures accounted for 55.3% (Table 2).

### 3.2. Medication Adherence

Patient medication adherence was measured and assessed through the GMAS scale. The adherence percentage was relatively low, at 57.6%

Low and poor adherence mainly were due to patient behavior (7.6% and 2.4%, respectively) (Table 3).

### 3.3. Associated Factors of Medication Adherence of Patients

Patients who lived in urban areas (OR = 1.336; 95%CI = 1.090–1.637; *p* = 0.005), who were employed (OR = 1.677; 95%CI = 1.251–2.248; *p* = 0.001), who had education levels at the level of high school or higher (OR = 1.313; 95%CI = 1.059–1.629; *p* = 0.013), or who had 0–1 comorbidities (OR = 1.293; 95%CI = 1.044–1.600; *p* = 0.019) were more likely to adhere to medications (*p* < 0.05). Patients who felt that the COVID-19 pandemic did not impact their willingness to continue their treatment and follow-up care had better medication adherence (OR = 1.771, 95% CI: 1.461–2.147). The proportion of medication adherence in patients applying 2 preventive measures or more was 1.422 times higher than in the other group (95% CI: 1.173–1.725) (Table 4).

## 4. Discussion

According to the medication adherence assessment using the GMAS scale, the proportion of medication adherence was relatively low (57.6%) and lower than the results of previous studies, with proportions of 61% and 68.1% [10,16]. The difference could be because the MMAS scale used in previous studies did not assess the influences of financial constraints on treatment. Moreover the research was conducted when the COVID-19 pandemic was spreading in Vietnam, leading to differences in the results.

Patients living in urban areas had a higher probability of medication adherence than patients living in rural areas (*p* = 0.005), which was similar to the previous study by Nouira et al. (2018) in Tunisia [10]. This could be because people in rural areas often have lower incomes than those in urban areas. Furthermore, the proportion of people with health insurance in rural areas is lower than that in urban areas [17]. Both of these reasons contributed to limiting patients’ ability to pay for healthcare treatment, negatively affecting medication adherence in patients [18].

The percentage of medication adherence for employed patients was higher than the figures for other groups (*p* = 0.001). The result differed from previous studies that did not find an association between work and medication adherence [19,20]. This difference can be explained by differences between the research subjects and job classification differences. In Vietnam, employees usually have their health insurance covered by their organization or by privileged healthcare preferential policies, which reduced the cost of drug treatment and increased the probability of medication adherence. The previous research by Schneider et al. (2018) also showed that health insurance or drug treatment cost assistance programs were factors that had a positive impact on medication adherence in patients [21].

Patients with higher education levels had a higher proportion of medication adherence (*p* = 0.013), similar to the study by Turcu-Stiolica et al. in Rome, from 2017 to 2019 [22]. Many of the results of previous studies demonstrated that people with poor health knowledge tended not to adhere to medication guidelines [23,24]. Achieving a high education level positively affects patient medication trust. Lemay et al. (2018) showed that patients with higher education levels also had positive beliefs about medication [25]. 

Patients with 0–1 comorbidity had higher medication adherence than patients with 2 or more comorbidities (*p* = 0.019). The results of previous studies also showed that people having multiple comorbidities and using multiple drugs at the same time often had a lower medication adherence [26,27]. This could be because patients with multiple comorbidities tend to use many drugs, making it challenging for them to manage their conditions and more likely to forget to use their medications more often. Patients using multiple drugs also had higher medication payments, which caused a lower probability of medication adherence than that of their counterparts [28]. 

Patients who felt that the COVID-19 pandemic impacted their willingness to continue treatment and follow-up care had a lower medication adherence score (*p* < 0.001), similar to those in the study of Hassen et al. (2020) [29]. One of the reasons why patients felt anxious was the fear of being infected with COVID-19 when going to crowded places [30,31]. Failure to attend follow-up care on time affected their medication adherence due to medication stoppage or changing the dose because of a shortage of medication or difficulties buying medicine because of economic conditions. 

The proportion of patients who applied 2 or more measures from the 5K message had higher medication adherence than the other groups. Kaye et al. (2020) also showed that using COVID-19 prevention guidelines positively affected treatment adherence [32]. In Vietnam, the measures from the 5K message were widely recommended through social media and other media, such as banners, posters, television, loudspeakers, etc. They were placed in many places for people to access quickly. Being aware of the COVID-19 pandemic has been shown to help increase the probability of complying with measures to prevent COVID-19 disease [33]. Therefore, it could be said that patients who applied more than 2 measures to avoid the COVID-19 pandemic had more concerns about their health problems, so they tended to use drugs more rationally. In addition, the application of measures to prevent the spread of the COVID-19 pandemic helped them both to protect themselves and to feel more secure when visiting outpatient clinics, ensuring their treatment and medical follow-ups proceeded according to the doctor’s instructions. 

We had some limitations in this study. The study was conducted when the COVID-19 pandemic was spreading in Vietnam to assess the disease’s impact on medication adherence. This might affect the accuracy of the recorded answers. Patients felt anxiety when interacting with strangers, so they might have answered quickly to keep distancing or might have refused to participate in the study. To overcome these obstacles, interviewers made sure to take good disease preventive measures, such as wearing masks, using hand sanitizer, and keeping a distance when interviewing subjects. At the same time, the interviewers also clearly explained the meaning of this study in terms of the subjects and the community. Secondly, researching the outpatient clinics did not sufficiently assess the impacts of the COVID-19 pandemic on patients with chronic diseases because it would miss patients who felt too anxious to go for re-treatment. Therefore, further studies should be conducted on a larger scale to enhance safety, to relieve patient anxiety during the COVID-19 pandemic, and to reach all the target subjects. In addition, measuring medication adherence using a questionnaire has some limitations.

To the best of the authors’ knowledge, to evaluate the effects of the COVID-19 pandemic on medication adherence among patients with chronic disease in Vietnam, it was timely and necessary to carry out this study during the complicated development of the COVID-19 pandemic. Our study found that the medication adherence proportion among patients during this period was relatively low. Therefore, it showed that basic characteristics such as living place, job, education level, number of comorbidities, anxiety about the COVID-19 pandemic, and the application of preventive measures according to the 5K message had significant influences on medication adherence in patients. This study makes it possible to conduct intervention studies to improve patient medication adherence and adjust medical examination and treatment practices during the COVID-19 pandemic.

The anxiety about the spread of the pandemic and the application of measures according to the 5K message were modifiable factors. Medical staff are the people who have the closest contact with patients, so healthcare providers must apply the 5K message and remind patients to take measures to protect themselves against the rapid spread of the pandemic, especially for patients with poor adherence, who often lack awareness of disease prevention. In addition to reducing the COVID-19 spreading, implementing an appropriate treatment approach is inevitably essential to enhance the management of medication adherence in patients with chronic diseases. Long-term drug delivery for patients with stable medical conditions and medication adherence management via phone or internet could be considered to achieve such goals. In addition, patients should be advised to adhere to medication guidelines and have better attitudes and practices on pandemic prevention to proactively protect their health, thus reducing complications related to chronic diseases, preventing the contracting of the COVID-19, and ensuring health.

## 5. Conclusions

The medication adherence of patients with chronic diseases was relatively low in southern Vietnam during the COVID-19 pandemic. Our research found some basic characteristics associated with medication adherence. It proved that patients who thought the COVID-19 pandemic obstructed treatment follow-up visits or who applied fewer than 2 measures for COVID-19 prevention were less likely to adhere to their medications. Healthcare providers should pay more attention to such patients and sufficiently perform preventive measures to reduce patient anxiety about the pandemic in order to achieve the desired treatment outcomes. Future studies might consider the identified factors in developing necessary interventions to improve the prevalence of medication adherence in the context of the complex and long-term COVID-19 pandemic.

## Figures and Tables

**Table 1:** Study population characteristics (*n* = 1852).

Characteristics	Frequency (n)	Percentage (%)
Age		
<60 years old	829	44.8
≥60 years old	1023	55.2
Mean ± standard deviation (years old): 60.12 ± 12.91		
Gender		
Male	841	45.4
Female	1011	54.6
Living place		
Rural areas	1032	55.7
Urban areas	820	44.3
Employment		
Employed ^a^	297	16.0
Unemployed ^b^	1555	84.0
Level of education		
Lower than high school	640	34.6
High school or higher	1212	65.4
Monthly income		
≤5 million VND	1051	56.7
>5 million VND	801	43.3
Chronic disease		
Digestive and hepatobiliary	109	5.9
Lung and respiratory	119	6.4
Endocrine	607	32.8
Cardiovascular	806	43.5
Musculoskeletal	139	7.5
Others	72	3.9
Duration of disease		
≤5 years	901	48.7
>5 years	951	51.3
Mean ± standard deviation (years): 7.43 ± 6.06		
Duration of treatment		
≤5 years	944	51.0
>5 years	908	49.0
Mean ± standard deviation (years): 7.21 ± 6.01		
Comorbidities		
0–1 comorbidities	1348	72.8
≥2 comorbidities	504	27.2
Mean ± standard deviation: 1.09 ± 0.99		

^a^ Being paid to work for a company or organization. ^b^ Retirement, running own business, i.e., without being paid.

**Table 2:** Attitudes and practices of patients during COVID-19 pandemic (*n* = 1852).

Variables	Frequency (n)	Percentage (%)
COVID-19 pandemic obstructed treatment follow-up visits		
Yes	1011	54.6
No	841	45.4
Feeling worried during visits		
Yes	1316	71.1
No	536	28.9
5K message		
Wearing facemask	1765	95.3
Disinfection	860	46.4
Distance	333	18.0
No gathering	237	12.8
Health declaration	307	16.6
Number of measures applied		
0–1 measures	828	44.7
≥2 measures	1024	55.3

**Table 3:** Characteristics of medication adherence according to the GMAS scale.

Medication Adherence	High *n* (%)	Good *n* (%)	Partial *n* (%)	Low *n* (%)	Poor *n* (%)
Non-adherence due to patient behavior	760 (41.0)	451 (24.4)	456 (24.6)	141 (7.6)	44 (2.4)
Non-adherence due to additional disease and pill burdens	951 (51.3)	503 (27.2)	324 (17.5)	53 (2.9)	21 (1.1)
Non-adherence due to financial constraints	984 (53.1)	416 (22.5)	400 (21.6)	28 (1.5)	24 (1.3)

**Table 4:** The relationship between the general characteristics and medication adherence of patients.

Variables	Medication Adherence (*n* = 1066)	Univariable Logistic Regression		Multivariable Logistic Regression	
	*n* (%)	OR (95% CI)	*p*	OR (95% CI)	*p*
Age					
≥60 years old	545 (53.3)	1	-	-	-
<60 years old	521 (62.8)	1.484 (1.231–1.788)	<0.001	-	-
Gender					
Female	574 (56.8)	1	-	-	-
Male	492 (58.5)	1.073 (0.892–1.292)	0.454	-	-
Living place					
Rural areas	542 (52.5)	1	-	1	-
Urban areas	524 (63.9)	1.6 (1.327–1.931)	<0.001	1.336 (1.090–1.637)	0.005
Employment					
Employed ^a^	851 (54.7)	1	-	1	-
Unemployed ^b^	215 (72.4)	2.169 (1.650–2.851)	<0.001	1.677 (1.251–2.248)	0.001
Level of education					
Lower than high school	310 (48.4)	1	-	1	-
High school or higher	756 (62.4)	1.765 (1.454–2.142)	<0.001	1.313 (1.059–1.629)	0.013
Monthly income					
≤5 million VND	554 (52.7)	1	-	-	-
>5 million VND	512 (63.9)	1.589 (1.317–1.919)	< 0.001	-	-
Comorbidities					
≥2 comorbidities	261 (51.8)	1	-	1	-
0–1 comorbidities	805 (59.7)	1.380 (1.123–1.696)	0.002	1.293 (1.044–1.600)	0.019
Duration of disease					
>5 years	520 (54.7)	1	-	-	-
≤5 years	546 (60.6)	1.275 (1.060–1.534)	0.010	-	-
Duration of treatment					
>5 years	501 (55.2)	1	-	-	-
≤5 years	565 (59.9)	1.211 (1.007–1.456)	0.042	-	-
COVID-19 pandemic obstructed treatment follow-up visits					
Yes	518 (51.2)	1	-	1	-
No	548 (65.2)	1.78 (1.475–2.148)	<0.001	1.771 (1.461–2.147)	<0.001
Feeling worried during visits					
Yes	720 (54.7)	1	-	-	-
No	346 (64.6)	1.507 (1.225–1.855)	<0.001	-	-
Number of measures applied					
0–1 measures	425 (51.3)	1	-	1	-
≥2 measures	641 (62.6)	1.587 (1.318–1.911)	<0.001	1.422 (1.173–1.725)	0.001

^a^ Being paid to work for a company or organization. ^b^ Retirement, running own business, i.e., without being paid.

## Data Availability

The datasets generated during and/or analyzed during the current study are available from the corresponding author on reasonable request.

## Appendix A

5K message of the Vietnamese Ministry of Health.

Vietnam Ministry of Health. The Ministry of Health recommends “5K” to live safely with the pandemic. Website about the evidence of the respiratory disease COVID-19. 2021. https://ncovmohgovvn/en/-/bo-y-te-khuyen-cao-5k-chung-song-an-toan-voi-dich-benh (accessed on 10 June 2021).

**Measure**	**Clarification**
Wearing facemask	Wear a facemask in public
Disinfection	Consists of frequent handwashing with soap or hand sanitizer, cleaning touched surfaces/items regularly (doorknobs, phones, tablets, tables, chairs, etc.), keeping the house clean, and spaces well ventilated
Distance	Keep a minimum distance of 2 m when communicating
No gathering	Do not gather in large numbers
Health declaration	Declare health conditions directly at neighboring medical facilities or via smartphone applications (Bluezone, COVID, and Vietnam Health Declaration).
